# Gelatin Reduced Graphene Oxide Nanosheets as Kartogenin Nanocarrier Induces Rat ADSCs Chondrogenic Differentiation Combining with Autophagy Modification

**DOI:** 10.3390/ma14051053

**Published:** 2021-02-24

**Authors:** Delong Jiao, Jing Wang, Wenting Yu, Ning Zhang, Ke Zhang, Yuxing Bai

**Affiliations:** 1 Institute of Dental Research, Beijing Stomatological Hospital & School of Stomatology, Capital Medical University, Beijing 100050, China; dianlan2@163.com; 2 Department of Orthodontics, Beijing Stomatological Hospital & School of Stomatology, Capital Medical University, Beijing 100050, China; kwwjjxa@163.com (J.W.); ortho_ywt@163.com (W.Y.); dentistzhang112@163.com (N.Z.); tuzizhangke@163.com (K.Z.)

**Keywords:** reduced graphene oxide, chondrogenic differentiation, kartogenin, adipose derived mesenchymal stem cell, autophagy

## Abstract

Biocompatible reduced graphene oxide (rGO) could deliver drugs for synergistically stimulating stem cells directed differentiation with influences on specific cellular activities. Here, we prepared a biodegradable gelatin reduced graphene oxide (rGO@Ge) to evaluate its functions in promoting rat adipose derived mesenchymal stem cells (ADSCs) chondrogenic differentiation through delivering kartogenin (KGN) into the stem cell efficiently. The optimum KGN concentration (approximately 1 μM) that promoted the proliferation and chondrogenic differentiation of ADSCs was clarified by a series of experiments, including immunofluorescent (IF) staining (Sox-9, Col II), alcian blue (Ab) staining, toluidine blue (Tb) staining and real-time quantitative PCR analysis of the chondrogenic markers. Meanwhile, the biocompatibility of rGO@Ge was evaluated to clearly define the nonhazardous concentration range, and the drug loading and releasing properties of rGO@Ge were tested with KGN for its further application in inducing ADSCs chondrogenic differentiation. Furthermore, the mechanism of rGO@Ge entering ADSCs was investigated by the different inhibitors that are involved in the endocytosis of the nanocarrier, and the degradation of the rGO@Ge in ADSCs was observed by transmission electron microscopy (TEM). The synergistic promoting effect of rGO@Ge nanocarrier on ADSCs chondrogenesis with KGN was also studied by the IF, Ab, Tb stainings and PCR analysis of the chondrogenic markers. Finally, the intracellular Reactive Oxygen Species (ROS) and autophagy induced by KGN/rGO@Ge complex composites were tested in details for clarification on the correlation between the autophagy and chondrogenesis in ADSCs induced by rGO@Ge. All the results show that rGO@Ge as a biocompatible nanocarrier can deliver KGN into ADSCs for exerting a pro-chondrogenic effect and assist the drug to promote ADSCs chondrogenesis synergistically through modification of the autophagy in vitro, which promised its further application in repairing cartilage defect in vivo.

## 1. Introduction

As a degenerative disease of the cartilage, osteoarthritis’ (OA) main pathological manifestations are the decomposition and fibrotic developments of cartilage extracellular matrix (ECM) resulting in structure and function loss of the articular cartilage [1]. The autologous cartilage grafts repair has a limited repair capacity due to the poor blood supply for chondrocyte proliferation and migration resulting in the unsatisfactory therapeutic effect [2]. With the development of cartilage engineering, the low regenerative capacity of chondrocytes in grafts restricts its application as the seed cell, so the mesenchymal stem cells (MSCs) have been introduced into the cartilage regeneration as a promising cell source with the ability of self-renewal and multiple differentiation potential [3].

The chondrogenesis of the MSCs usually needs the inductive agents, such as the transforming growth factor β3 (TGFβ3), which is a principal factor in cartilage development. However, the over-dose usage of TGFβ3 would cause side-effects, including osteophyte, inflammation and fibroplasia [4]. Recently, kartogenin (KGN) as a small molecule drug has been proved to facilitate human mesenchymal stem cells (HMSCs) chondrogenesis in a dose-dependent manner (median effective concentration (EC50): 100 nM) [5], and it provides us with the safer choice of the chondro-inducers that act on MSCs to promote chondrogenesis with less side-effects [6]. Among the choices of the seed cells, the adipose derived mesenchymal stem cells (ADSCs) is considered a good MSCs candidate for cartilage regeneration, because its harvesting process is less invasive and a great number of cells can be acquired by expanding in vitro culture simply [7]. The research on the efficient drug delivery strategy would have a great significance in achieving the optimum ADSCs chondrogenesis induced by KGN in cartilage repair.

In recent years, the graphene-based materials show a potential biomedical applications with a good biocompatibility due to its unique structure and geometry characters [8]. Graphene oxide (GO), as the single-atom-thick layer of graphene sheet with carboxylate, hydroxyl and epoxide functional groups, can adsorb the drugs and biomolecules through π–π interactions [9] and also function as filler to modify the hydrogel for better biological functions [10,11]. Reduced graphene oxide (rGO) is acquired through reducing GO via high-temperature thermal treatment or chemical reducing agents treatment which provided it with more functional groups [12]. Both GO and rGO have been reported to be the nanocarrier for delivering the drug molecules to induce stem cell differentiation [13,14,15]. Due to the poor solubility and unstable dispersion of GO, the different modification methods were applied to induce the covalent or noncovalent functional groups for the improvement of solubility and stability [16,17,18]. The reduction methods of synthesizing biocompatible rGO from GO were developed with more choices of biological reducing reagents to modify the nanocarrier for broader and safer biological application [19,20]. Gelatin as the hydrolyzed collagen is a reducing polypeptide, in which the amino acids on its molecule chain can be oxidized to nitrite [21]. Our previous synthesized gelatin reduced graphene oxide (rGO@Ge) has been verified as a biocompatible nanocarrier for promoting osteogenesis of the bone marrow mesenchymal stem cells (BMSCs) synergistically with the osteo-inductive drug [22]. The promised nanocarrier function of rGO@Ge prospects its potential application in delivering KGN for promoting ADSCs chondrogenesis.

With regard to the drug delivery strategy, researchers discovered that the distributions of the nanocarrier in the stem cell could influence its drug delivery function and exert a specific effect on cellular life activities, which resulted in the regulation of stem cell differentiation and proliferation [23]. Autophagy as the cellular homeostasis mechanism could remove the dysfunctional organelles and macromolecules, and recent observations illustrate that the pathogenesis of the aging-related diseases, including OA, usually involves the change of compromised or defective autophagy [24]. It has also been reported that the chondrogenesis of MSCs could be promoted via the modulation of autophagy [25]. This indicates that the augmentation of the cellular homeostasis mechanism (autophagy) may be a new strategy for the modification of the MSCs chondrogenesis in OA treatment. So, in our research, we would firstly clarify the effects of rGO@Ge on the KGN delivery into ADSCs for chondrogenesis and further investigate the entry pathways and degradation of rGO@Ge in ADSCs. Furthermore, the change of autophagy in ADSCs was also evaluated to define the relationship between the chondrogenesis and autophagy modulated by rGO@Ge. Through the serials experiments, we would sketch the possible mechanism of rGO@Ge on the drug delivery and synergistic pro-chondrogenic differentiation effect. To our knowledge, our study is the first to evaluate the correlation between the rGO@Ge-induced autophagy and its promotive effect on MSCs chondrogenesis in vitro. The detailed research about effects of rGO@Ge on promoting ADSCs chondrogenesis with KGN would propose its promising application in the cartilage regeneration repair in vivo.

## 2. Materials and Methods

### 2.1. Rat ADSCs Isolation and Culture

Adipose derived mesenchymal stem cells (ADSCs) were isolated from 4-week-old male Sprague-Dawley (SD) rats. The inguinal adipose tissue was collected from the SD rats under sterilized condition and rinsed by phosphate buffer saline (PBS) three times. The tissue block was cut into pieces with microsurgical scissors and digested with collagenase type I (1 mg/mL, Sigma-Aldrich, USA) for 60 min at 37 °C, then the digestion product was vortexed for further dispersion. The mixture solution was centrifuged with 1500 rpm for 10 min, the supernatant was discarded and the remaining pellet was resuspended with the Dulbecco’s modified Eagle medium (DMEM) (supplemented with 10% Foetal Bovine Serum (FBS) and 1% penicillin/streptomycin) for the cell expansion culture in a humidified atmosphere of 5% CO_2_ in air at 37 °C. When the cell colonies became large and confluent, ADSCs were digested by trypsin for passage with medium changes every 3 days. Subsequently, ADSCs acquired from passage 2 to 3 were used in our research. All animal experiments were approved by the Animal Ethics and Welfare Committee of Beijing Stomatological Hospital & School of Stomatology, Capital Medical University (KQYY-202008-003).

### 2.2. The Effects of KGN on Rat ADSCs

Before the investigation on the rGO@Ge, the chondrogenic differentiation ability of ADSCs was first studied using the DMEM medium supplemented with 100 nM dexamethasone (Sigma-Aldrich), 1% insulin-transferrin-selenium (Sigma-Aldrich), 50 μM ascorbate-2-phosphate, 1 mM sodium pyruvate, 50 mg/mL Bovine Serum Albumin (BSA), 5 μg/mL linoleic acid (Sigma-Aldrich), 1% penicillin/streptomycin and with 10 ng/mL TGFβ3 (R&D Systems) or different concentrations of KGN (Sigma-Aldrich). The medium containing TGFβ3 was used to verify the chondrogenic differentiation ability of rat ADSCs, and the concentration gradient of KGN in the DMEM medium was 100 nM, 500 nM, 1 μM and 10 μM. For evaluation on ADSCs proliferation under different concentrations of KGN, the cells were seeded in a 96-well plate with a density of 4 × 10^3^ per well and tested by Cell Counting Kit-8 (CCK8) assays according to manufacturer’s protocol at 1, 4 and 7 days. After incubation with the detection reagent at 37 °C for 1 h, the acquired optical density (OD) value at 450 nm was used to reflect the proliferation ability of ADSCs. The chondrogenic differentiation of ADSCs was further tested by PCR, immunofluorescent (IF) staining, alcian blue (Ab) and toluidine blue (Tb) stainings. ADSCs were seeded in a 24-well plate with a density of 1 × 10^5^ per well and the medium containing different KGN concentrations was used to culture cell for evaluation on ADSCs chondrogenesis. The culture medium containing KGN was changed after the first cell seeding 24 h and refreshed every other day in the subsequent experiments. After 14 days culture, the chondrogenic differentiation of ADSCs was terminated and the cell was fixed using 4% paraformaldehyde (PFA) for the subsequent IF (Sox-9 and Col II), Ab and Tb stainings. Meanwhile, the total RNA was extracted for the PCR analysis of the chondrogenic makers (Sox-9, Col II, Aggrecan and Col X), as in the previously described process [22]. The procedures of the staining and PCR analysis were also briefly described in subsequent sections.

### 2.3. Synthesis of rGO@Ge and Evaluation on Its Nanocarrier Function

The gelatin reduced graphene oxide (rGO@Ge) was synthesized as our previous study [22]. Here is a brief description of the method: graphene oxide (GO) was chemically reduced with excessive gelatin through stirring for 24 h at 95 °C, and the resulting reaction solution was centrifuged at 20,000 rpm with hot water washing three times for removing excess gelatin. The synthesized rGO@Ge was dissolved in water and stored at 4 °C until use. The drug loading–releasing property of rGO@Ge was tested with kartogenin (KGN). The 5 μL KGN solution (1 mM) was mixed with different amounts of 100 mg/mL rGO@Ge solution (the amounts were 0, 2.5, 5, 7.5, 10, 12.5, 15, 17.5, 20, 22.5 and 25 μL) in a total 300 μL volume of PBS solution. The mixture system was incubated at room temperature on a shaking table for 2 h and centrifuged with 14,000 rpm for 10 min so as to monitor the free KGN concentration in the supernatant. Through calculation, the loading capacity of rGO@Ge on KGN can be clarified and the nanocarrier with saturable absorption of KGN (rGO@Ge/KGN) was collected for further analysis of the releasing kinetic curve. The 30 mg rGO@Ge/KGN was used to test the releasing of KGN with different pH (7, 5 and 2) in 1mL PBS or acetate buffer solution at 37 °C, and the KGN concentration in the supernatant after centrifugation was tested after shaking for different times (1, 3, 5, 7, 9, 12, 24, 36, 48, 60, 72, 96, 120, 144, 168 and 264 h). The KGN has a characteristic absorption peak at 280 nm in the UV-vis spectrum, and the OD value is linearly related KGN concentration, which provides us with a reliable method for quantitative research on the loading–releasing kinetics.

### 2.4. The Biocompatibility and Entry Pathways of rGO@Ge in ADSCs

The biological effects of rGO@Ge on ADSCs were tested by live/dead staining, CCK8 assay and flow cytometry analysis so as to evaluate its cytotoxicity and influences on proliferation and apoptosis of ADSCs, respectively. The concentration series of rGO@Ge were 0, 10, 10^2^, 10^3^ and 10^4^ μg/mL in the DMEM medium (supplemented with 10% FBS and 1% penicillin/streptomycin). Calcein-AM/PI staining kit (Yeasen, Shanghai, China) and Annexin V-FITC/PI staining kit (Thermo Fisher Scientific, USA) were used for material cytotoxicity and cell apoptosis analysis. The proliferation of ADSCs in the rGO@Ge medium was tested at 1, 4 and 7 days by CCK8 as described above. The rGO@Ge was labeled with Rhodamine 6G (R6G) as previously described [22], and ADSCs were incubated with medium containing R6G-labeled rGO@Ge for observation of the nanocarrier distribution in the cell. Through pretreating ADSCs with different endocytosis inhibitors, the amount change of rGO@Ge that entered into cell was studied by flow cytometry quantitative analysis to clarify the main entry pathway of rGO@Ge in ADSCs. The inhibitors included wortmannin (30 μM), amiloride (1 mM) and genistein (40 μM) that inhibited phagocytosis, micropinocytosis and caveolae-dependent endocytosis, respectively [26]. After the co-culture of ADSCs and rGO@Ge for 24 h, the collected samples were prepared as the ultrathin sections (70 nm) using ultramicrotome (Leica EM UC7), and the transmission electron microscope (TEM) (H-7650; Hitachi, Tokyo, Japan) was used to observe the location and degradation of rGO@Ge in ADSCs.

### 2.5. ADSCs Chondrogenesis Induced by KGN-Loaded rGO@Ge

After clarification on the biocompatibility and nanocarrier function of rGO@Ge, the studies on the application of KGN-loaded rGO@Ge in promoting ADSCs chondrogenesis were further carried out to evaluate the functions of the drug-nanocarrier complex composites. The concentration of KGN in the composites was set to 1 μM, and the different concentrations of rGO@Ge were mixed with KGN, which composed the different medium with a series of KGN/rGO@Ge concentration ratios (1 μM KGN corresponding to 0, 10, 10^2^, 10^3^ and 10^4^ μg/mL rGO@Ge). For the convenience of description to the different groups, we abbreviated these KGN/rGO@Ge ratios to K/G values, so the above described series medium were named as K/G0 (1 μM KGN: 0 μg/mL rGO@Ge), K/G1 (1 μM KGN: 10 μg/mL rGO@Ge), K/G2 (1 μM KGN: 10^2^ μg/mL rGO@Ge), K/G3 (1 μM KGN: 10^3^ μg/mL rGO@Ge) and K/G4 (1 μM KGN: 10^4^ μg/mL rGO@Ge) in short. The ADSCs were seeded in a 24-well plate with a density of 1 × 10^5^ per well and the different medium with K/G values was used to culture ADSCs for evaluation on the stem cell chondrogenesis. After 14 days of cell culture, the IF, Ab and Tb stainings were carried out to analyze the chondrogenic differentiation of ADSCs. Meanwhile, PCR was also conducted to evaluate the ADSCs chondrogenesis.

### 2.6. Analysis on the Intracellular ROS and Autophagy Changes in ADSCs Treated with Different K/Gs Media

The intracellular Reactive Oxygen Species (ROS) in ADSCs was measured using the ROS Assay Kit (Yeasen, Shanghai, China). The ADSCs were cultured with different K/Gs media for 24 h and the medium containing 2,7-Dichlorodi-hydrofluorescein diacetate (DCFH-DA) fluorescent probe was used to replace the different K/Gs media for detecting the ROS levels in different groups according to the manufacturer’s protocol. After 30 min of incubation with the fluorescent probe, the ADSCs were washed with PBS three times for the subsequent fluorescent microscope imaging and flow cytometry quantitative analysis. To explore the change of autophagy in ADSCs that was induced by the KGN/rGO@Ge composites, the K/G0, 1, 2, 3 and 4 series media were also used to treat the ADSCs for 24 h and the incubated cells were fixed with 4% PFA for the LC3B IF staining to evaluate the autophagy activity. In the meantime, parallel experiments were also carried out to verify the changes of autophagy-related protein expression in ADSCs for autophagy analysis. To determine the role of the Erk1/2 signal pathway in the pro-autophagy effect of rGO@Ge and the role of autophagy in the pro-chondrogenic effect of rGO@Ge, the Erk1/2 signal pathway inhibitor PD98059 (CST, USA) and autophagy inhibitor 3-methyladenine (3-MA; Selleck, USA) were added into the K/G3 medium for the evaluation on the changes of autophagy and chondrogenesis in ADSCs. During the chondrogenesis of ADSCs that were treated with K/G3 medium containing PD98059 or 3-MA, the culture medium was changed every 3 days with the addition of the Erk1/2 signal pathway and autophagy inhibitors.

### 2.7. Immunofluorescence (IF), Alcian Blue (Ab) and Toluidine Blue (Tb) Stainings

As for the immunofluorescence staining, the fixed cell samples were firstly washed with PBS three times and then incubated with 0.5% Triton X-100 with 5 min for membrane rupture. The cells were blocked with 10% BSA for 1 h and incubated overnight with different primary antibodies at 4 °C according to the corresponding experiments, including anti-LC3B antibody (Abcam, USA), anti-Sox-9 antibody (Abcam, USA) and anti-Col II antibody (Abcam, USA). Then, the samples were washed with PBST (PBS with 0.05% Tween-20) three times and incubated with the secondary antibodies. Subsequently, the nucleus was stained with 4′,6-diamidino-2-phenylindole (DAPI) and the cytoskeleton was stained with FITC-labeled phalloidin. The images of the IF staining were acquired by the confocal microscope and fluorescent microscope. The chondrogenic differentiation of ADSC samples were also stained with alcian blue and toluidine blue staining solutions (Sangon Biotech, Shanghai, China) for 30 min, washed with PBS and observed under the light microscope. The propanol dissociated solution was used to dissolve the alcian blue dye, and the OD value at 600 nm was detected for quantitative analysis of the glycosaminoglycan (GAG) content in the Ab stained samples.

### 2.8. Real-Time Quantitative PCR Analysis

Firstly, the total RNA was extracted from the ADSCs chondrogenesis samples with TRIzol reagent (Life Technologies, USA) according to the manufacturer’s protocol. Subsequently, the RNA was reverse transcribed into the complementary DNA (cDNA) using the reverse transcription kit (Takara, Japan). At last, the real-time quantitative PCR was conducted using SYBR Green Master Mix (Roche, USA) by ROCHE480 real-time PCR system to detect the changes of chondrogenic makers, including Sox-9, Col II, Aggrecan and Col X. The sequences of primers for each gene were listed in Table 1. The tested genes were normalized using β-actin as the internal reference. The expression level of each gene was analyzed using the comparative Ct (2^−∆∆Ct^).

### 2.9. Western Blotting Analysis

The cell samples were lysed by Radio-Immunoprecipitation Assay (RIPA) Buffer (Beyotime, China) containing phosphatase inhibitor cocktail and phenylmethylsulfonyl fluoride (PMSF) for protein extraction. The total cell proteins were denatured with SDS-PAGE loading buffer, separated by SDS-PAGE gel and transferred to Polyvinylidene Fluoride (PVDF) membrane (Millipore, USA). Then the membranes were blocked with 10% BSA and incubated with primary antibodies including anti-ULK1 (Abcam, USA), anti-Beclin-1 (Abcam, USA), anti-LC3 (Abcam, USA), p-Erk1/2 (CST, USA), Erk1/2 (CST, USA) and anti-β-actin (CST, USA) overnight at 4 °C. Subsequently, the blots were washed with TBST (Tris Buffered Saline with 0.05% Tween 20) three times and incubated with the Horseradish Peroxidase (HRP) conjugated secondary antibodies (1:10000, CST) for 45 min. The chemiluminescence reagent (Thermo, USA) was used to expose and identify the protein bands using ChemiDoc Touch System (Bio-Rad, USA), and quantitative analysis of the protein expression level was calculated relative to the β-actin control using the ImageJ software (NIH, USA).

### 2.10. Statistical Analysis

All the experiments were repeated three times and the quantitative data were expressed as mean ± SD. The statistical analysis of multiple comparisons was performed using a one-way ANOVA and Student–Newman–Keul tests with the SPSS 13.0 software (SPSS Inc., USA). *p* values < 0.05 were considered statistically significant.

## 3. Results

### 3.1. Promotion of ADSCs Chondrogenesis by KGN

**Figure 1:**
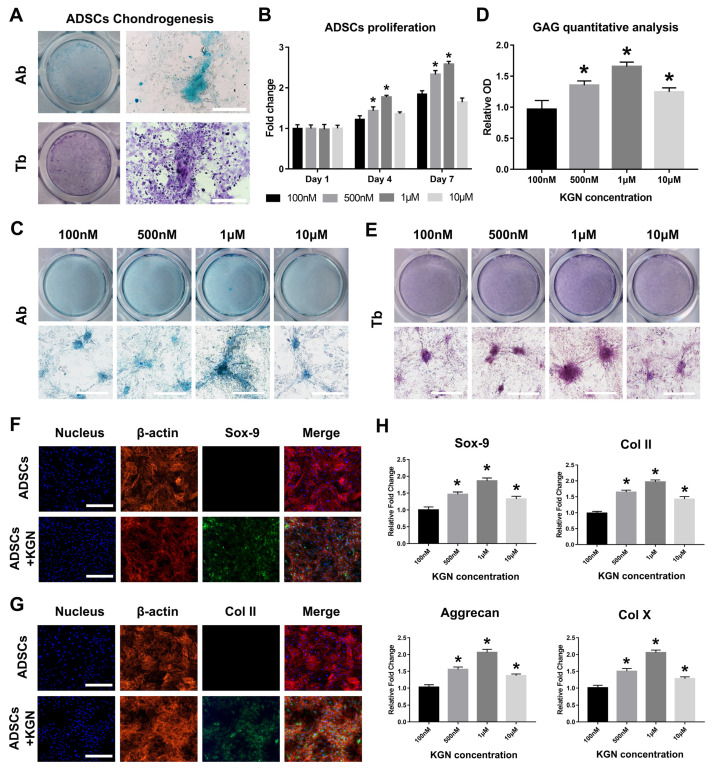
The chondrogenic differentiation of adipose derived mesenchymal stem cells (ADSCs) induced by kartogenin (KGN). (**A**) The verification of ADSCs chondrogenesis by transforming growth factor β3 (TGFβ3) and the Ab and Tb stainings showed the successful chondrogenic differentiation, Bar: 200 μm. (**B**) the Cell Counting Kit-8 (CCK8) analysis of the ADSCs proliferation treated with different KGN concentrations. * *p* < 0.05 indicates a significant difference compared to the corresponding 100 nM group at each time point. (**C**) The evaluation of different KGN concentrations on ADSC chondrogenic differentiation. The Ab stainings were used to test the effects of different KGN concentrations (100 nM, 500 nM, 1 μM and 10 μM), bar: 200 μm. (**D**) The quantitative analysis of the alcian blue staining to evaluate the glycosaminoglycan (GAG) content. * *p* < 0.05 indicates a significant difference compared to the 100 nM group. (**E**) The evaluation of different KGN concentrations on ADSCs chondrogenic differentiation. The Tb stainings were used to test the effects of different KGN concentrations (100 nM, 500 nM, 1 μM and 10 μM), bar: 200 μm. Immunofluorescence staining of the chondrogenic markers Sox-9 (**F**) and Col II (**G**) to clarify the ADSCs chondrogenesis, Bar: 200 μm. (**H**) Real-time PCR quantitative analysis of the chondrogenic markers including Sox-9, Col II, Aggrecan and Col X. * *p* < 0.05 indicates a significant difference compared to the 100 nM group.

The chondrogenic differentiation capacity of ADSCs was verified through culturing with traditional chondrogenic inductive medium containing 10 ng/mL TGFβ3 for 14 days. The alcian bule (Ab) and toluidine blue (Tb) stainings (Figure 1A) showed the ADSCs chondrogenic differentiation induced by TGFβ3, the glycosaminoglycan (GAG) in the cartilage matrix nodule was stained with alcian blue and the secreted chondroitin sulfate was also stained with toluidine blue. All the results prove that the cultured ADSCs had differentiated into chondrocytes. After the verification of the ADSCs chondrogenesis, we next evaluated the effects of different KGN concentrations on ADSCs proliferation and chondrogenic differentiation. The CCK8 analysis (Figure 1B) indicated that the 1 μM KGN had the best promoting effect on cell proliferation and the results of Ab (Figure 1C) and Tb (Figure 1E) stainings show that the medium containing 1 μM KGN also had the optimum effect on promoting ADSCs chondrogenesis. Meanwhile, the glycosaminoglycan (GAG) quantitative analysis of the alcian blue staining (Figure 1D) also showed the best ADSCs chondrogenesis induced by 1 μM KGN. For further clarification of the ADSCs chondrogenesis induced by 1 μM KGN, the immunofluorescence staining of Sox-9 and Col II (Figure 1F,G) showed the enhanced expression of the chondrogenic markers induced by KGN. The real-time PCR quantitative analysis of the chondrogenic markers (Figure 1H) reflected the influence of KGN concentration on the ADSCs chondrogenesis, and the Sox-9, Col II, Aggrecan and Col X all showed the similar expression pattern induced by series of KGN medium. We also compared the effects of TGFβ3 and KGN on rat ADSCs chondrogenesis (Figure S1A,B), which showed that the 1 μM KGN had the equivalent pro-chondrogenic differentiation effect on ADSCs compared with 10 ng/mL TGFβ3. All of the above results indicate that the ADSCs induced by 1 μM KGN had the best chondrogenic differentiation capacity.

### 3.2. Nanocarrier Function and Biocompatibility of rGO@Ge

**Figure 2:**
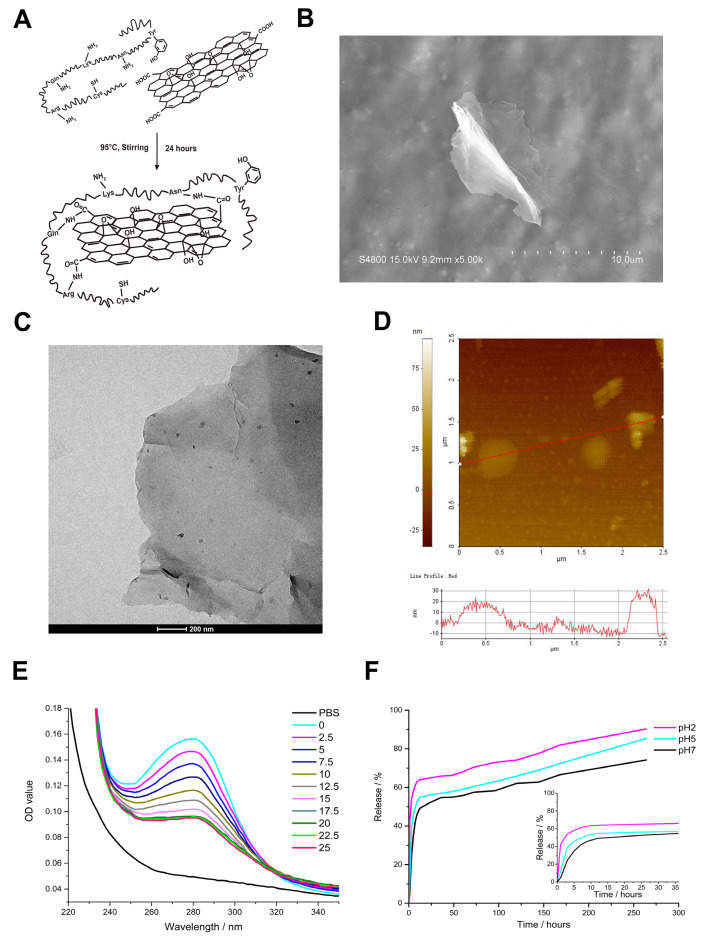
The nanocarrier function of gelatin reduced graphene oxide (rGO@Ge). (**A**) The schematic diagram of the rGO@Ge synthesis. The scanning electron microscopy (SEM) (**B**), transmission electron microscopy (TEM) (**C**) and atomic force microscopy (AFM) (reprinted with permission from Ref. [22]. Copyright 2021 American Chemical Society). (**D**) detections showed the surface morphology of rGO@Ge. (reprinted with permission from Ref. [22]. Copyright 2021 American Chemical Society). (**E**) The loading curves of the different amounts of rGO@Ge mixed with the same amount of KGN. (**F**) The KGN releasing kinetic curves of the KGN-loaded rGO@Ge in different pH media.

The brief description of the rGO@Ge synthesis was shown in a schematic diagram (Figure 2A). The glutamine (Gln), arginine (Arg) and asparagine (Asn) as the reducing amino acids could react with GO to construct the rGO@Ge. The detailed characterization analysis of rGO@Ge had been reported in our previous research [22], and the surface morphology was characterized with scanning electron microscopy (SEM), transmission electron microscopy (TEM) and atomic force microscopy (AFM) (Figure 2B–D). The detection on the size distribution of the rGO@Ge (Figure S1C) showed that the particle size of rGO@Ge nanocarrier was around 150 nm. The gelatin attached on the rGO@Ge could guarantee its nanocarrier function and better biocompatibility, and the KGN loading curves showed that the KGN concentration would reach a stable low level with the addition of increased rGO@Ge. Through calculation with the OD value at 280 nm, the rGO@Ge loading capacity of KGN could be obtained as 1mg rGO@Ge, corresponding to 2 nM KGN (Figure 2E). The releasing kinetic curve of KGN-loaded rGO@Ge in different pH media (Figure 2F) showed the burst release of KGN in the first 12 h, and the acidic conditions could accelerate the KGN release speed and amount. After a long time, the total releasing amount of KGN in different pH media also showed a big difference with a maximum releasing amount of approximately 90% in pH 2 medium, which facilitated the KGN release in the acidic phase of the OA lesion region in the early stage of drug delivery by the nanocarrier.

**Figure 3:**
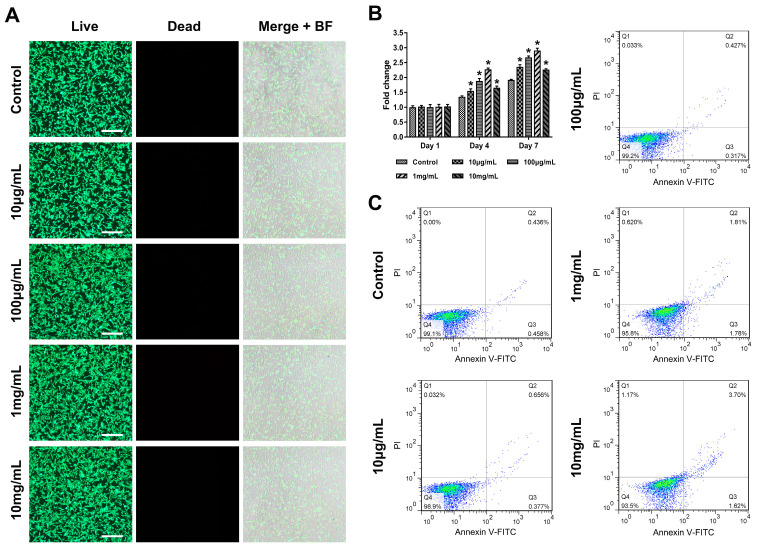
The biocompatibility of rGO@Ge. (**A**) The live/dead staining of ADSCs cultured with different rGO@Ge concentration media, Merge+BF: the merge of Live, Dead and Background Field images, Bar: 500 μm. (**B**) CCK8 assay on the ADSCs proliferation at 1, 4 and 7 days. * *p* < 0.05 indicates a significant difference compared to the corresponding control group at each time point. (**C**) Flow cytometry analysis of the ADSCs apoptosis with the different rGO@Ge concentration stimulus.

The biocompatibility of rGO@Ge tested by live/dead staining (Figure 3A) illustrated its low cytotoxicity with the non-detection of dead ADSCs. Meanwhile, the CCK8 analysis of the ADSCs proliferation suggested (Figure 3B) that the 1 mg/mL rGO@Ge medium showed the best promotion effect on ADSCs proliferation. The flow cytometry analysis of the apoptosis (Figure 3C) showed that ADSCs had an increased apoptotic rate with the increased rGO@Ge concentration due to the stimulus of the nanomaterials. The above data imply that the optimum rGO@Ge concentration was at approximately 1 mg/mL, which had the best promotion effect on cell proliferation without excess cell apoptosis.

### 3.3. The Entry Pathway and Degradation of rGO@Ge in ADSCs

**Figure 4:**
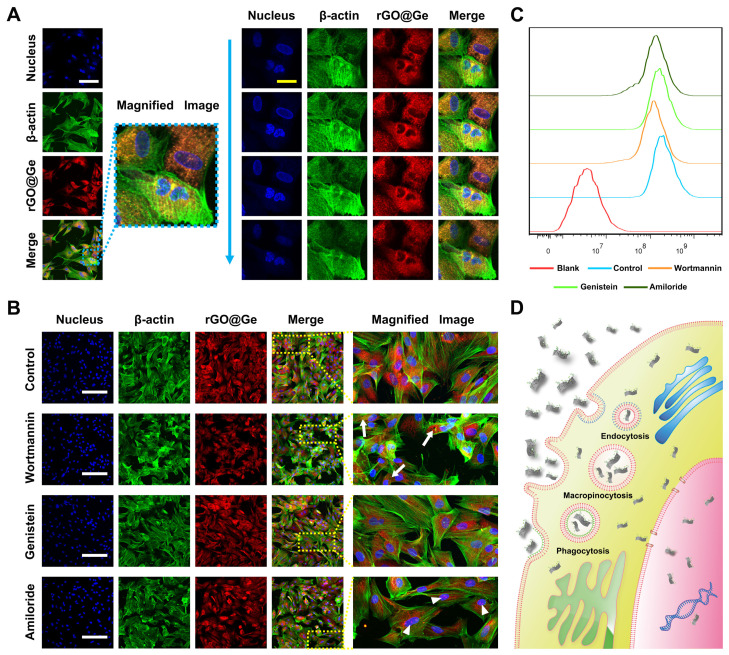
The entry pathway of rGO@Ge in ADSCs. (**A**) The Rhodamine 6G (R6G)-labeled rGO@Ge was taken up by ADSCs and it was located in the cytoplasm and nucleus; the right part shows the different images via the vertical Z axis, white bar: 100 μm, yellow bar: 25 μm. (**B**) The changes of the rGO@Ge taken up by ADSCs that were pretreated with different inhibitors. The arrow indicates the vacuole due to the blocky of the phagocytosis by wortmannin, and the arrowhead indicates the accumulation of rGO@Ge in the nucleus due to the inhibition of macropinocytosis by amiloride, bar: 200 μm. (**C**) The flow cytometry analysis on the quantity variance of the R6G-labeled rGO@Ge that were taken up by ADSCs between the different inhibitor-pretreated groups. (**D**) Schematic diagram illustrating the pathway of rGO@Ge entering ADSCs and its distributions in cell.

The confocal images of the R6G-labled rGO@Ge treated ADSCs (Figure 4A) showed the widespread distributions of rGO@Ge in the cytoplasm and nucleus. The serial sections in the vertical Z axis illustrated the detailed location of the nanomaterial in the nucleus, which indicated its promised nanocarrier function for drug delivery. The ADSCs were pretreated with different endocytic mechanism inhibitors so as to explore the primary entry pathway of rGO@Ge (Figure 4B), the wortmannin inhibited the phagocytosis of the nanomaterials showing the small vacuoles without R6G-labeled rGO@Ge, genistein inhibited the caveolae-dependent endocytosis with a slightly decreased amount of rGO@Ge distributed in ADSCs and amiloride inhibited the macropinocytosis showing the obvious decrease of rGO@Ge in the cytoplasm combined with a particulate aggregation in the nucleus. The fluorescence activated cell sorting (FACS) analysis on the fluorescent intensity of R6G-labled rGO@Ge (Figure 4C) showed the quantitative differentials between the different inhibitor-treated groups, the wortmannin-treated ADSCs showed the least rGO@Ge content, the amiloride group ranked the second in the decreased rGO@Ge endocytic amount and the genistein treated group did not show a distinct difference to the control group.

**Figure 5:**
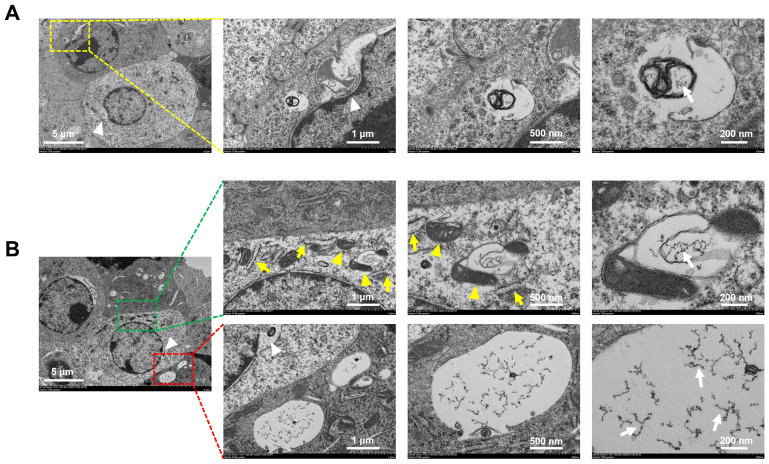
The location and degradation of rGO@Ge in ADSCs. (**A**) Transmission electron microscope (TEM) showed the location of rGO@Ge around the nucleus (white arrowhead) and the potential degradation of rGO@Ge as a small fragment (white arrow). (**B**) The location of rGO@Ge in the cytoplasm was adjacent to mitochondria (yellow arrowhead) and rough endoplasmic reticulum (yellow arrow), and the potential degradation residue of rGO@Ge was observed as a small fragment (white arrow).

The schematic image (Figure 4D) presented the entry pathway and the distributions of rGO@Ge in ADSCs. The TEM (Figure 5) also showed the widespread distributions of the rGO@Ge in ADSCs; its location around the nucleus (white arrowhead in Figure 5A,B) illustrated its nucleus entry process. In the cytoplasm, the rGO@Ge was adjacent to mitochondria (yellow arrowhead in Figure 5B) and rough endoplasmic reticulum (yellow arrow in Figure 5B). The maximum magnification field of the rGO@Ge in ADSCs showed the different degradation stages of the nanosheets in the cell, and the potential degradation residue could be observed as some small fragments (white arrow in Figure 5) in the TEM. The biodegradation of the rGO@Ge in ADSCs promised its application as a safe and reliable nanocarrier.

### 3.4. Promotion of ADSCs Chondrogenesis by KGN-Loaded rGO@Ge

**Figure 6:**
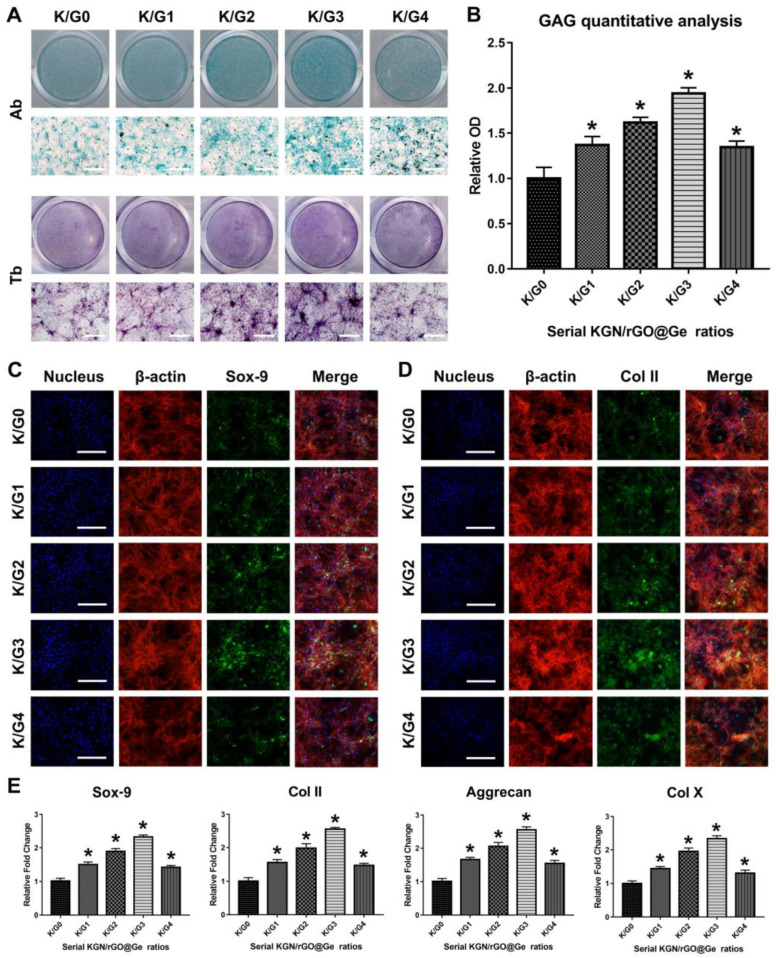
The chondrogenic differentiation of ADSCs induced by KGN/rGO@Ge complex media. (**A**) The evaluation of different K/G ratio media on the ADSCs chondrogenic differentiation. The Ab and Tb stainings were used to test the effects of different K/Gs (1 μM KGN corresponding to 0, 10, 10^2^, 10^3^ and 10^4^ μg/mL rGO@Ge) medium, bar: 500 μm. (**B**) The quantitative analysis of the alcian blue staining to evaluate the GAG content. * *p* < 0.05 indicates a significant difference compared to the K/G0 group. Immunofluorescence staining of the chondrogenic differentiation marker Sox-9 (**C**) and Col II (**D**) to clarify the ADSCs chondrogenesis, Bar: 200 μm. (**E**) The PCR analysis on the expression of the chondrogenic differentiation markers in ADSCs cultured with the different K/G media. * *p* < 0.05 indicates a significant difference compared to the K/G0 group.

The ADSCs chondrogenic differentiation was tested by alcian blue (Ab) and toluidine blue (Tb) stainings (Figure 6A) so as to evaluate the synergetic effects of different KGN/rGO@Ge ratio composites on the ADSCs chondrogenesis. The results of the Ab and Tb stainings show that the K/G3 group exerted the optimal promotion effect on ADSCs chondrogenic differentiation. The high concentration of rGO@Ge had a reverse effect on the stem cell chondrogenesis, and the best fit ratio of the K/Gs was 1 μM KGN corresponding to 10^3^ μg/mL rGO@Ge nanocarrier (K/G3). The GAG quantitative analysis of the Ab staining (Figure 6B) also showed the best chondrogenic inductive effect of the K/G3 group. The IF staining of the chondrogenic differentiation marker Sox-9 (Figure 6C) and Col II (Figure 6D) illustrated the similar trend with the Ab and Tb stainings, the K/G3 group exerted the best promotion effect on the expression of the chondrogenic makers. The PCR analysis (Figure 6E) indicated that the chondrogenic differentiation markers, including Sox-9, Col II, Aggrecan and Col X, all showed the first rising and then declining trend with the increased rGO@Ge concentration. All the results show that the K/G3 group had the best chondrogenic inductive effect and the over-high rGO@Ge concentration was harmful to the ADSCs chondrogenesis.

### 3.5. The Effects of K/Gs on Intracellular ROS and Autophagy of ADSCs Correlated to Chondrogenesis

**Figure 7:**
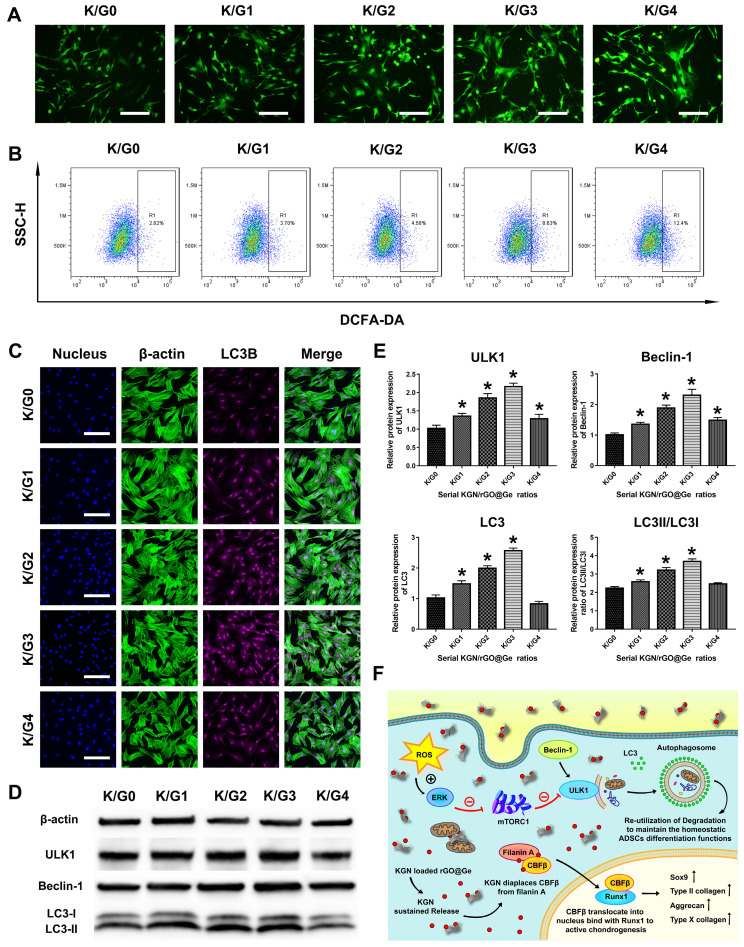
The change of the autophagy activity in ADSCs cultured with KGN/rGO@Ge medium. (**A**) The fluorescent microscope images of the intracellular Reactive Oxygen Species (ROS) in the different K/Gs media treated ADSCs, bar: 200 μm. (**B**) The flow cytometry analysis on the intracellular ROS in the ADSCs treated with the different K/Gs media. R1 gated the quantitative changes of the intracellular ROS in the different groups. (**C**) The LC3B immunofluorescent staining was used to test the autophagic activities, bar: 200 μm. (**D**) The Western blot analysis on the autophagic proteins to reflect the autophagy level in ADSCs. The ULK1, Beclin-1, and LC3 were all expressed most in the K/G3 group. (**E**) The quantitative analysis of the ULK1, Beclin-1, LC3, and LC3II/LC3I. * *p* < 0.05 indicates a significant difference compared to the K/G0 group. (**F**) The illustration of the mechanism that KGN/rGO@Ge induced the ADSCs chondrogenic differentiation as the nanocarrier for delivering KGN and regulating autophagy activity. The ROS activated the Erk1/2 signal pathway, then the mammalian target of rapamycin complex 1 (mTORC1) was inhibited and the mammalian target of rapamycin (mTOR) within mTORC1 was also inhibited, so the complex of mTOR and ULK1 would disassemble and the dissociated ULK1 from mTOR further initiated the autophagy.

The intracellular ROS fluorescent images (Figure 7A) showed that the increased amount of the rGO@Ge aroused the ROS accumulation in ADSCs treated with K/Gs media for 24 h. The quantitative analysis by flow cytometry (Figure 7B) also illustrated the continuous ROS increasement due to the increasement of rGO@Ge, and the K/G4 group had the most detection of intracellular ROS in the R1 gate by approximately 12.4%. However, the LC3B immunofluorescent staining (Figure 7C) in ADSCs that was cultured with K/Gs media for 24 h, showed the highest expression level in the K/G3 group. The proper rGO@Ge concentration could cause the gradual rise of autophagy in a range below 1 mg/mL, while the high rGO@Ge dose 10 mg/mL induced the decrease of the autophagy activity due to the over-high ROS damage, which resulted in the worst chondrogenesis (Figure 6) and the most apoptosis (Figure 3C). The Western blotting analysis of the autophagic proteins (Figure 7D) also showed the same expression pattern with the LC3B IF staining, and the ULK1, Beclin-1, and LC3 proteins had the highest expression level in the K/G3 group, which had the best promotion effect on the ADSCs chondrogenesis. The quantitative analysis of the autophagy-related proteins (Figure 7E) also showed the highest LC3II/LC3I ratio in the K/G3 group, which indicated that more LC3II participated in autophagosome formation to activate the autophagy activity.

**Figure 8:**
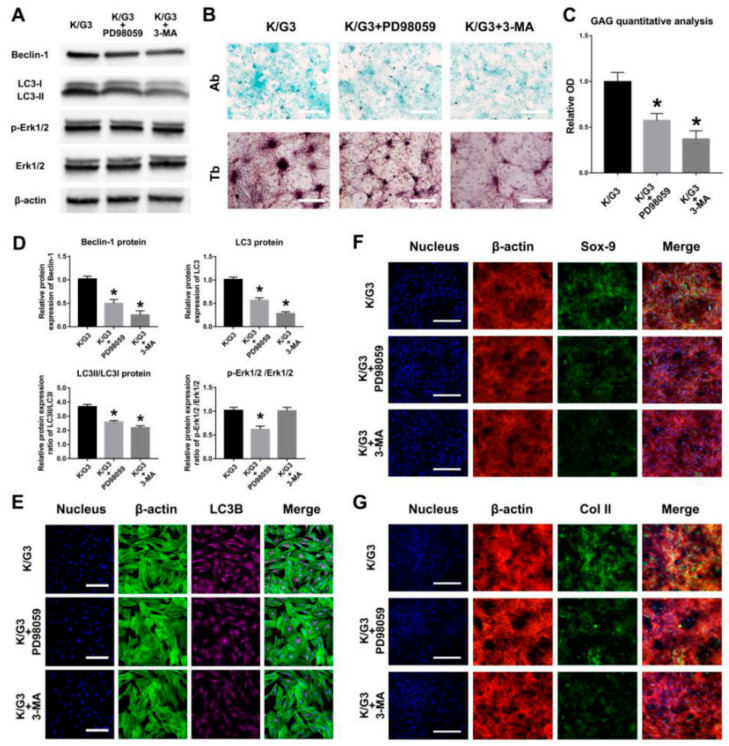
The correlation between the autophagy and chondrogenesis induced by rGO@Ge in ADSCs cultured with K/G3 medium. (**A**) The Western blot analysis on the Erk1/2 signal pathway and autophagy associated proteins in ADSCs co-cultured with PD98059 or 3-MA in K/G3 medium. (**B**) The Ab and Tb stainings of the ADSCs chondrogenesis treated with K/G3, K/G3+PD98059 and K/G3+3-MA medium, bar: 500 μm. (**C**) The quantitative analysis of the alcian blue staining to evaluate the GAG content. * *p* < 0.05 indicates a significant difference compared to the K/G3 group. (**D**) The quantitative analysis of the Beclin-1, LC3, LC3II/LC3I, p-Erk1/2 /Erk1/2. * *p* < 0.05 indicates a significant difference compared to the K/G3 group. (**E**) The LC3B immunofluorescent staining was used to test the autophagic activities, bar: 200 μm. Immunofluorescence staining of the chondrogenic markers Sox-9 (**F**) and Col II (**G**) clarified the difference of ADSCs chondrogenesis in three groups, Bar: 200 μm.

The schematic picture (Figure 7F) illustrated the possible mechanism of the KGN/rGO@Ge composites promoting ADSCs chondrogenesis. The rGO@Ge as the nanocarrier could deliver the KGN into ADSCs efficiently and the biocompatible effective dose also could activate the autophagy to regulate the cellular differentiation behavior. To clarify the role of the Erk1/2 signal pathway and autophagy in the rGO@Ge induced chondrogenesis, we added PD98059 and 3-MA into K/G3 medium for analysis on ADSCs chondrogenic differentiation. The Western blot results (Figure 8A,D) show that the inhibition of the Erk1/2 signal pathway could cause a partial decrease of autophagic proteins, and 3-MA could inhibit the autophagy activity to a relatively low level, which indicated that the Erk1/2 signal pathway was activated to induce autophagy by rGO@Ge. Furthermore, the Ab and Tb stainings (Figure 8B) showed the differences of ADSCs chondrogenesis among the different groups, and we could observe that the inhibited autophagy (K/G3+PD98059 and K/G3+3-MA) caused the retardant ADSCs chondrogenesis compared with the K/G3 group. The GAG quantitative analysis of the alcian blue staining (Figure 8C) also showed the effect of autophagy on ADSCs chondrogenesis. The IF staining of LC3B (Figure 8E) showed the inhibited autophagy level in the PD98059 and 3-MA treated group compared with the K/G3 group. Meanwhile, the IF stainings of Sox-9 (Figure 8F) and Col II (Figure 8G) showed the consistent decreased expression patterns, which clarified the correlation between the autophagy and chondrogenesis induced by rGO@Ge.

All of the above results show that the proper KGN/rGO@Ge ratio composite could synergistically amplify the pro-chondrogenic effect of KGN via autophagy modification in vitro, which promised its further application in the cartilage regeneration repair in vivo.

## 4. Discussion

In our study, we utilized the rat ADSCs as the mesenchymal stem cell to evaluate the effects of KGN and its rGO@Ge complex on MSCs chondrogenic differentiation. The results concerning the ADSCs chondrogenesis induced by KGN illustrate that the efficient drug concentration was 1 μM, and the proper concentration of the drug could promote the ADSCs proliferation and chondrogenic differentiation. These observations were consistent with the recent studies about the effect of KGN on other MSCs chondrogenic differentiation, such as umbilical cord mesenchymal stem cells [27] and bone marrow mesenchymal stem cells [28]. The decreased proliferation ratio and chondrogenic gene expressions in the 10 μM KGN group were detected in our study, which potentially resulted from the over-differentiation of ADSCs into hypertrophic chondrocytes with less formation of the cartilage matrix and expression of chondrogenic genes under the induction of the exorbitant KGN. It has also been reported that KGN at low concentrations was beneficial to cell proliferation and the over-high KGN concentrations would promote cell differentiation with decreased promotion on proliferation [29]. The successful chondrogenesis of rat ADSCs also provided more experimental evidence for its promised seed cell function in cartilage regeneration repair. Meanwhile, the detection of the Sox-9 and Col II chondrogenic markers in the KGN induced ADSCs in our study certified its chondrogenesis potential as a stem cell, so ADSCs could be used as the seed cell for the subsequent experiments about the rGO@Ge biological functions. The PCR analysis also supported the ADSCs chondrogenesis potential with a promised application in cartilage regeneration repair.

The rGO@Ge as the reduced graphene oxide (rGO) had the essential properties of rGO, in which more functional groups on the rGO nanosheet surface could extend its application in biomedical fields including neural [30], cardiac [31] and bone [32] tissue engineering. Compared with the synthetic substrate GO, the gelatin on the surface of rGO@Ge guaranteed it the better stability in different media as described in our previous study [22]. Meanwhile, the biomacromolecule gelatin attached on rGO@Ge also prospects its promised biocompatibility and potential biodegradation compared with the un-modified GO. The further analysis on its function as nanocarrier for KGN delivery in our study showed its widespread locations in the cytoplasm and nucleus of ADSCs, which guaranteed its efficient delivery of the KGN molecule into the MSCs to exert its promoting chondrogenic differentiation effect. The live/dead staining illustrated the good biocompatibility of rGO@Ge even at a high dose of 10 mg/mL, whereas its promotion effect on ADSCs proliferation had attained the optimum efficiency at 1 mg/mL with a decreased cell viability at 10 mg/mL. The analysis on the ADSCs apoptosis influenced by different rGO@Ge concentrations showed the great increased apoptotic ratio of ADSCs at 10 mg/mL, which explained the decrease of cell proliferation ratio due to the high dose of rGO@Ge. The analysis about the entry pathways of rGO@Ge showed that ADSCs take in the nanocarrier mainly via phagocytosis and macropinocytosis mechanisms. The wortmannin-inhibited ADSCs showed the small vacuoles without R6G-labeled rGO@Ge, and the FACS analysis also showed the significant decrease of R6G fluorescence intensity. The inhibited macropinocytosis by amiloride also elicited a decreased uptake of rGO@Ge by ADSCs, and the flow cytometry illustrated the less quantitative difference with the control compared with the wortmannin-treated group. The genistein inhibited endocytosis pathway had less effect on the rGO@Ge’s entry into ADSCs. The clarification about the mechanism of the rGO@Ge entry pathways provided us with more information about its nanocarrier function for drug molecules delivery. The TEM analysis of the rGO@Ge in ADSCs also showed its widespread locations, and its closer distribution to the nucleus might have favored its synergistic effect on chondrogenesis with efficient KGN delivery. Meanwhile, the potential small fragment form of the rGO@Ge degradation residue was similar to the Nitric Oxide (NO)-dependent biodegradation of GO reported recently [33]. Meanwhile, the rGO could also induce the formation of NO [34], which potentially explains the biocompatibility and biodegradation of rGO@Ge and showed clearer tracks of rGO@Ge nanocarrier in ADSCs. The previous studies about the mechanism of the graphene materials in cancer treatment discovered the mitochondria targeting and oxidative stress phenomenon [35,36]. In our study, the TEM also showed that the location of rGO@Ge was adjacent to mitochondria and rough endoplasmic reticulum, which implied the possible mechanism of the nanomaterials in causing ROS production. The proper ROS level caused by rGO@Ge in the ADSCs would further influence the cellular behaviors and the chondrogenic differentiation capacity of the MSCs.

The tests about the pro-chondrogenic effect of the KGN/rGO@Ge composites on ADSCs showed the synergistic effect of rGO@Ge with KGN on promoting chondrogenesis. The K/G3 group as the best efficient complex composite was composed of 1 μM KGN concentration and 1 mg/mL rGO@Ge. So, in the K/G3 group, 1 mL complex medium contained 1 nM KGN and 1 mg rGO@Ge, which had the same order of magnitude as the rGO@Ge loading capacity of KGN (2 nM KGN loaded by 1 mg rGO@Ge). The IF staining of chondrogenic differentiation markers including Sox-9 and Col II, the alcian blue and toluidine blue stainings and the PCR analysis on the chondrogenic relevant markers all illustrated the optimum efficiency of the K/G3 group in ADSCs chondrogenesis. The previous studies reported that the graphene-based materials could cause the reactive oxygen species (ROS) production to exert its biological functions on the cellular behaviors [37,38], and the ROS in the cytoplasm was shown to be an essential regulator of the autophagy activity in the cartilage homeostasis mechanisms [24]. Recent studies also showed that the regulation of autophagy could have a chondro-protective effect on osteoarthritis therapy and cartilage repair [39,40]. In our research, we also observed the ROS triggered by rGO@Ge, and the autophagy activity could be influenced by the different rGO@Ge concentrations. The increased ROS caused by the dose increasement of nanomaterial elicited a regulating effect on the autophagy, the gradual increased autophagy with rGO@Ge dose increasement in a relatively low concentration range guaranteed a promised pro-chondrogenic effect and the over-high nanomaterial concentration would cause a reverse effect on the ADSCs chondrogenesis due to the excessive ROS damage. The over-high rGO@Ge concentration caused severe ADSCs apoptosis, and the autophagy activity was also inhibited abnormally. All of the changes led to the decreased ADSCs chondrogenesis compared with the differentiation potential induced by a proper nanomaterial concentration range (10~1000 μg/mL). The worst cellular behaviors and chondrogenesis caused by over-high rGO@Ge implied that the proper nanomaterial concentrations needed be selected carefully so as to reserve its good stimulus function and avoid the severe damage for exerting its favorable biological effects on MSCs. Meanwhile, the ROS/ERK and mTOR signaling pathway was also previously reported as the mechanism of the autophagy modification [41], and we also observed that the Erk1/2 signal pathway was activated in the rGO@Ge-induced augmentation of autophagy, which indicated the possible mechanism of rGO@Ge on autophagy modification.

The above results of the KGN/rGO@Ge composites show us the synergistic effects of the nanocarrier with KGN for promoting ADSCs chondrogenesis through autophagy modification in vitro. So, the proper complex of KGN and rGO@Ge would have a promised application in cartilage repair in vivo and the animal OA model experiments were needed to conduct for the further research.

## 5. Conclusions

In our study, the gelatin reduced graphene oxide showed the good biocompatibility and nanocarrier function for the load–release of KGN. The 1 μM KGN was verified to have the optimum pro-chondrogenic effect, and the proper KGN/rGO@Ge composite (1 μM KGN: 1 mg/mL rGO@Ge) could enhance ADSCs chondrogenesis in a synergistic manner. The ROS induced by rGO@Ge guaranteed the nanocarrier an extended biological function to exert a series of effects on cellular behaviors such as proliferation, apoptosis and autophagy modifications. At last, we can confirm that the KGN/rGO@Ge composite could promote rat ADSCs chondrogenesis efficiently in vitro and potentially has a promised application in cartilage regeneration repair in vivo.

## Figures and Tables

**Table 1:** Primer sequences used in qRT-PCR analyses.

Gene Name	Forward and Reverse Sequence
Sox-9-F	5′-TCAACGGCTCCAGCAAGAACAAG-3′
Sox-9-R	5′-CTCCGCCTCCTCCACGAAGG-3′
Col II-F	5′-ACGCTCAAGTCGCTGAACAACC-3′
Col II-R	5′-ATCCAGTAGTCTCCGCTCTTCCAC-3′
Aggrecan-F	5′-CTGATCCACTGTCCAAGCACCATG-3′
Aggrecan-R	5′-ATCCACGCCAGGCTCCACTC-3′
Col X-F	5′-TGATCCTGGAGTGGGAGGAG-3′
Col X-R	5′-GGGATACCTGGTGGTCCAAT-3′
β-actin-F	5′-GTAAAGACCTCTATGCCAACA-3′
β-actin-R	5′-GGACTCATCGTACTCCTGCT-3′

**Abbreviations:** F, Forward sequence; R, Reverse sequence.

## Data Availability

The data presented in this study are available on request from the corresponding author. The data are not publicly available due to privacy.

## Supplementary Materials

The following are available online at https://www.mdpi.com/1996-1944/14/5/1053/s1, Figure S1: The comparison on the chondrogenic differentiation of ADSCs induced by TGFβ3 or KGN, and the particle size distribution of rGO@Ge. (A) The Ab and Tb stainings showed the similar chondrogenic differentiation of ADSCs in both 10 ng/mL TGFβ3 and 1 μM KGN culture medium. (B) The quantitative analysis of the alcian blue staining to evaluate the GAG content, which indicates no significant difference between two groups. (C) The particle size of rGO@Ge was distributed around 150 nm.
