# Supplementary material for: Gelatin Reduced Graphene Oxide Nanosheets as Kartogenin Nanocarrier Induces Rat ADSCs Chondrogenic Differentiation Combining with Autophagy Modification
Source: Materials (Basel). 2021 Feb 24;14(5):1053. doi: 10.3390/ma14051053 (PMC7956601; doi:10.3390/ma14051053)
Supplement: Supplementary file 1 [file materials-14-01053-s001.pdf]

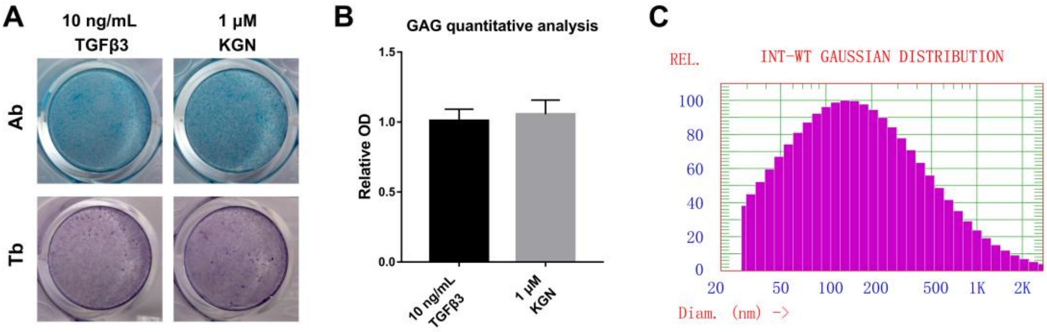

Figure S1. The comparison on the chondrogenic differentiation of ADSCs induced by TGFβ3 or KGN, and the particle size distribution of rGO@Ge. (**A**) The Ab and Tb stainings showed the similar chondrogenic differentiation of ADSCs in both 10 ng/mL TGFβ3 and 1 μM KGN culture medium. (**B**) The quantitative analysis of the alcian blue staining to evaluate the GAG content, which indicates no significant difference between two groups. (**C**) The particle size of rGO@Ge was distributed around 150 nm [1].

References

1 Jiao, D.; Zheng, A.; Liu, Y.; Zhang, X.; Wang, X.; Wu, J.; She, W.; Lv, K.; Cao, L.; Jiang, X. Bidirectional differentiation of BMSCs induced by a biomimetic procallus based on a gelatin-reduced graphene oxide reinforced hydrogel for rapid bone regeneration. *Bioact. Materials* **2021**, *6*, 2011–2028.
